# Stress Alters the Discriminative Stimulus and Response Rate Effects of Cocaine Differentially in Lewis and Fischer Inbred Rats

**DOI:** 10.3390/bs2010023

**Published:** 2012-03-01

**Authors:** Therese A. Kosten, Mindy J. D. Miserendino

**Affiliations:** 1 Menninger Department of Psychiatry and Behavioral Sciences, Baylor College of Medicine and the Michael E Debakey Veteran’s Administration Medical Center, 2002 Holcombe Blvd., Houston, TX 77030, USA; 2 Department of Psychology, Sacred Heart University, 5151 Park Ave, Fairfield, CT 06825, USA; E-Mail: MiserendinoM@sacredheart.edu

**Keywords:** drug discrimination, HPA axis, corticosterone, inbred rat strain, addiction

## Abstract

Stress enhances the behavioral effects of cocaine, perhaps via hypothalamic-pituitary-adrenal (HPA) axis activity. Yet, compared to Fischer 344 (F344) rats, Lewis rats have hyporesponsive HPA axis function and more readily acquire cocaine self-administration. We hypothesized that stress would differentially affect cocaine behaviors in these strains. The effects of three stressors on the discriminative stimulus and response rate effects of cocaine were investigated. Rats of both strains were trained to discriminate cocaine (10 mg/kg) from saline using a two-lever, food-reinforced (FR10) procedure. Immediately prior to cumulative dose (1, 3, 10 mg/kg cocaine) test sessions, rats were restrained for 15-min, had 15-min of footshock in a distinct context, or were placed in the shock-paired context. Another set of F344 and Lewis rats were tested similarly except they received vehicle injections to test if stress substituted for cocaine. Most vehicle-tested rats failed to respond after stressor exposures. Among cocaine-tested rats, restraint stress enhanced cocaine’s discriminative stimulus effects in F344 rats. Shock and shock-context increased response rates in Lewis rats. Stress-induced increases in corticosterone levels showed strain differences but did not correlate with behavior. These data suggest that the behavioral effects of cocaine can be differentially affected by stress in a strain-selective manner.

## 1. Introduction

Stress enhances the behavioral effects of psychostimulant drugs such as cocaine [1,2,3]. Acute exposure to stressors increases the place conditioning effects of stimulants [4], and promotes acquisition [5,6,7,8,9] and reinstatement [10,11] of stimulant self-administration. This interaction has important implications for vulnerability to drug addiction as well as relapse to use after abstinence. Indeed, vulnerability and relapse to drug use after abstinence are associated with stressful life events in drug addicts [12,13,14] and medications that reduce stress hormone responses may help prevent stress-induced relapse in addicts [15].

The ability of stress to enhance the behavioral effects of cocaine may reflect additive effects on the hypothalamic-pituitary–adrenal (HPA) system. Like stressor exposure, cocaine activates the HPA axis [16,17,18,19,20,21]. Alternatively, the enhancement of cocaine’s effects by stress may reflect additive effects of mesocorticolimbic dopamine system activity that plays an important role in subserving the psychomotor, motivational and/or reinforcing properties of cocaine [22,23,24]. Stress enhances dopaminergic activity in this system [2,25,26]. 

HPA axis function, mesolimbic dopamine system activity, and behavioral responses to cocaine differ between Lewis and Fischer (F344) inbred rats [17]. Compared to F344 rats, Lewis rats have hyporesponsive HPA axis function [27,28,29], show decreased responses to stressors [30,31,32], and exhibit evidence of decreased mesolimbic dopamine function [33,34,35]. Yet, Lewis rats display enhanced acquisition of cocaine place conditioning [36] and self-administration [37] compared to F344 rats, consistent with results seen with other psychoactive drugs [17]. To date, there are no studies that have examined the effects of acute stress on the behavioral effects of cocaine in these inbred strains. Previous research in other rat strains and in mice shows that the discriminative stimulus effects of psychoactive drugs generalize to [38,39] and are enhanced by [40,41] acute stressor exposure. The present study sought to determine whether there are strain-selective effects of acute stress on the discriminative stimulus and response rate effects of cocaine in Lewis and F344 inbred rats. 

## 2. Results and Discussion

There were no strain differences in numbers of trials necessary to reach the criterion for acquisition of the cocaine-saline discrimination. Cocaine discrimination was acquired in about 24 sessions (range 16–42 sessions). 

### 2.1. Baseline Vehicle and Cocaine Discrimination Tests

#### 2.1.1. Vehicle Test in Cocaine Test Groups

A vehicle discrimination test was performed for the cocaine test groups. In this test, rats were injected with the saline vehicle at the three time segments to be used in the cumulative cocaine dose procedure. Data obtained from this 3-segment vehicle test demonstrate that both Lewis and F344 rats show primarily vehicle-appropriate responding (*i.e.*, very low levels of cocaine-appropriate responding) as seen in Table 1. There are no significant effects of Strain or Segment on percent cocaine-appropriate responding (P’s > 0.10). Response rates during the 3-segment vehicle session are greater in Lewis *vs.* F344 rats, F(1,14) = 32.51; P < 0.0001 as seen in Table 1. Response rates do not differ by segment (P > 0.10). 

**Table 1:** *Baseline Vehicle Test in Cocaine Test Groups*. Mean (± S.E.M.) percent cocaine-appropriate responding and response rates (total lever presses/sec) during a cumulative dosing session with vehicle injections in F344 and Lewis rats in the cocaine test groups.

*Segment*	*% Cocaine-appropriate responding*		*Response rate*	
	*F344 rats*	*Lewis rats*	*F344 rats*	*Lewis rats*
1	0.7 (0.7)	11.3 (7.6)	0.68 (0.06)	1.08 (0.07)
2	1.9 (1.0)	2.5 (1.3)	0.73 (0.06)	1.02 (0.08)
3	15.0 (11.0)	0.5 (0.5)	0.73 (0.06)	0.03)

#### 2.1.2. Vehicle Test in Vehicle Test Groups

Data obtained from the 3-segment vehicle test for vehicle test groups demonstrate that both Lewis and F344 rats show primarily vehicle-appropriate responding as seen in Table 2. There are no significant effects of Strain or Segment effects on percent cocaine-appropriate responding (Ps > 0.10). Response rates during the 3-segment vehicle test are greater in Lewis *vs.* F344 rats, F(1,8) = 21.97; P < 0.01 as seen in Table 2. Response rates do not differ by segment (P > 0.10). 

**Table 2:** *Baseline Vehicle Test in Vehicle Test Groups*. Mean (± S.E.M.) percent cocaine-appropriate responding and response rates (total lever presses/sec) during a cumulative dosing session with vehicle injections in F344 and Lewis rats in the vehicle test groups.

*Segment*	*% Cocaine-appropriate responding*		*Response rate*	
	*F344 rats*	*Lewis rats*	*F344 rats*	*Lewis rats*
1	1.7 (6.1)	0.6 (0.2)	0.47 (0.06)	1.14 (0.14)
2	1.3 (5.9)	1.6 (0.8)	0.47 (0.06)	0.67 (0.19)
3	0 (3.6)	0	0.31 (0.04)	0.85 (0.03)

#### 2.1.3. Cocaine Test in Cocaine Test Groups

**Figure 1:**
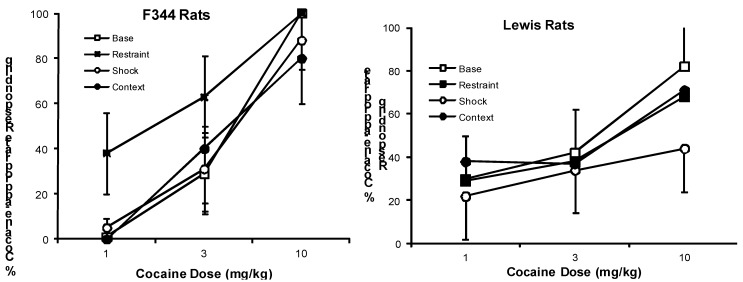
Mean (± S.E.M.) percentage of responding on the cocaine-appropriate lever by cocaine dose (mg/kg) is shown for F344 (left panel) and Lewis (right panel) rats under baseline (open squares) conditions and following exposure to restraint (closed squares), footshock (open circles), and footshock-associated context (closed circles). Restraint enhanced the discriminative stimulus effects of cocaine in F344, but not in Lewis rats. Other stressors were without effect in both strains.

The Cocaine test groups were then tested for discrimination with cocaine injections using the cumulative dosing procedure. Under this baseline condition with cocaine injections, percent cocaine-appropriate responding increases with increasing cocaine dose, F(2,134) = 17.55; P<.0001. As seen in Figure 1, Lewis rats show a somewhat flatter dose-response curve compared to F344 rats, although the Strain effect is not significant. Data obtained from this cocaine baseline test were compared to the baseline session with vehicle injections. There are significant Drug, F(1,14) = 46.35; P < 0.0001, Segment, F(2,28) = 13.64; P < 0.001, and Drug X Segment, F(2,28) = 11.72; P < 0.005 effects on cocaine-appropriate responding, but no significant Strain effect (P>0.10). Comparing response rates in the cocaine baseline test to the baseline session with vehicle injections showed a significant effect of Strain, F(1,14) = 41.80; P < 0.0001 and a trend towards significance for Drug, F(1,14)3.37; P < 0.10. 

### 2.2. Effects of Stressors on Discrimination Performance

#### 2.2.1. Cocaine Test Groups

Once it was established that rats in both strains showed mainly vehicle-appropriate responding during the vehicle test and mainly cocaine-appropriate responding during the cocaine test, tests were begun with the three stressors (restraint, shock, and shock context). There is a significant interaction of Condition (Baseline, shock, restraint, context) × Dose × Strain, F(6,134) = 2.08; P = 0.05. Separate analysis by strain reveals that restraint produces an increase in cocaine-appropriate responding in F344, F(1,69) = 5.26; P < 0.05, but not in Lewis rats, P > 0.10. Neither footshock nor the context paired with it significantly affects cocaine-appropriate responding in either strain (Ps > 0.10). There is no significant effect of the order of presentation (P > 0.10). These data are shown in Figure 1.

#### 2.2.2. Vehicle Test Groups

In general, exposure to stressors leads to total cessation of responding in most rats in the vehicle test groups. For the shock test, only one Lewis rat and no F344 rat completed at least one response requirement. Only two Lewis rats and one F344 rat completed at least one response requirement after the context test. Thus, it is not possible to examine the effects of these stressors on cocaine discrimination behavior. We are able to examine effects of restraint stress on responding in a limited manner as five Lewis rats and three F344 rats completed at least one response requirement during the first test segment. Responding ceased in all F344 rats and in most Lewis rats during the second and third test segments. Thus, for the restraint stress test, only the discrimination data obtained from the first test segments of the baseline *vs.* the restraint test session are compared. Restraint stress increases cocaine-appropriate responding compared to the baseline condition (see Figure 1) in both F344 (33.0 ± 10.3%) and Lewis (37.9 ± 17.3 %) rats. This statement is supported by the significant Condition effect, F(1,17) = 6.34; P < 0.05. There is no significant strain difference (P > 0.10).

### 2.3. Effects of Stressors on Response Rates

#### 2.3.1. Cocaine Test Groups

**Figure 2:**
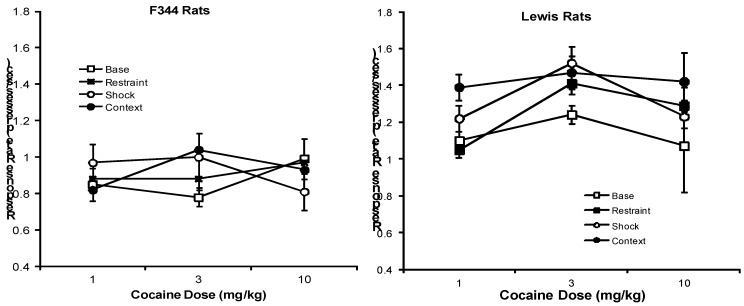
Mean (± S.E.M.) response rates (number of lever presses/sec) by cocaine dose (mg/kg) is shown for F344 (left panel) and Lewis (right panel) rats under baseline (open squares) conditions and following exposure to restraint (closed squares), footshock (open circles), and footshock-associated context (closed circles). Both footshock and its associated context increased response rates to cocaine in Lewis, but not in F344 rats. Restraint stress was without effect in both strains.

A different picture emerges when the effects of these stressors on response rates are examined in the cocaine test groups. There are significant effects of Condition, F(3,134) = 6.01; P < 0.001, and Strain, F(1,134) = 69.99; p < 0.0001, and a trend towards significance for the interaction of Condition × Strain, F(3,134) = 2.25; P < 0.09. Subsequent separate analyses by strain show that both footshock and its context enhance response rates in Lewis rats above baseline levels, F’s(1,69) = 5.03; 5.26; Ps < 0.05, respectively. As seen in Figure 2, none of the stressors altered response rates in F344 rats (Ps > 0.10).

#### 2.3.2. Vehicle Test Groups

As described above, we could only examine the effects of restraint stress during the first test segment on response rates. Restraint stress decreases response rates compared to the baseline condition in both F344 (0.24 ± 0.10 presses/sec) and Lewis (0.80 ± 0.09 presses/sec) rats. This is supported by the significant Condition effect, F(1,17) = 6.94; P < 0.05. Also, Lewis rats show significantly higher response rates than F344 rats, F(1,17) = 27.42; P < 0.0001. This strain effect is seen under both baseline and restraint stress conditions. Thus, strain does not differentially alter the effects of restraint on response rates as evidenced by the lack of significance for the interaction Strain X Condition effect (P > 0.10). 

### 2.4. Corticosterone Levels

Corticosterone samples were obtained from seven Lewis and six F344 rats, all of them from the cocaine test groups. Cocaine administration (10 mg/kg; IP) and exposure to each stressor increase corticosterone compared to baseline levels as seen in Table 3. This is supported by the significant main effect of Condition, F(4,44) = 33.23; P < 0.0001, as well as a Condition x Strain interaction, F(4,44) = 9.60; P < 0.0001. Separate analyses (simple effects) by strain reveal no significant strain differences in corticosterone levels at baseline or following cocaine administration although somewhat higher baseline levels are seen in F344 rats (see Table 3). Lewis rats show greater corticosterone levels following shock, F(1,11) = 12.68; P < 0.005, while F344 rats show a tendency toward greater corticosterone levels in response to restraint stress, F(1,11) = 4.22; P < 0.07, and significantly greater corticosterone levels in response to the shock context, F(1,11) = 6.18; P < 0.05. Corticosterone levels do not correlate with either behavioral measure under any condition, (P’s > 0.10).

**Table 3:** *Corticosterone levels after cocaine and stress exposures*. Mean (± S.E.M.) corticosterone (ng/mL) levels obtained after exposure to the training dose of cocaine and to the stressors in F344 and Lewis rats.

*Condition*	*F344*	*Lewis*
Baseline	249 (50)	187 (20)
Cocaine	519 (57)	646 (46)
Restraint	367 (33)	220 (45)
Shock*	455 (58)	714 (45)
Exposure to shock context*	516 (28)	368 (40)

* denotes significant strain differences.

## 3. Experimental Section

### 3.1. Subjects

Adult male Fischer 344 (F344) and Lewis rats (Harlan, Indianapolis, Ind., USA) weighing approximately 300–320 g (F344) or 310–350 g (Lewis) were used in this study. Rats were group-housed, three to a cage, in hanging wire-mesh cages in a temperature-controlled colony room with a 12:12 hr light/dark cycle (lights on at 7:00 a.m.). Animal facilities were accredited by the American Association for the Accreditation of Laboratory Animal Care. Food (Purina Rat Chow) and tap water were available *ad libitum* unless otherwise noted. Training and test procedures were conducted between 1200 and 1500 hr. Procedures were approved by the Institutional Animal Care and Use Committee and followed the “Principles of Laboratory Animal Care” (NIH publication No. 85-23, revised 1996).

### 3.2. Groups

One set each of Lewis and F344 rats (n = 8 each) was employed in the cocaine test groups. Another set each of Lewis (n = 7) and F344 rats (n = 6) was employed in the vehicle test groups. All rats were trained to discriminate cocaine as described below. Test sessions with the cocaine test groups were conducted with cocaine administration and tested the interaction of stress and cocaine on cocaine discrimination. The vehicle test groups were used to test whether stress alone substituted for the cocaine cue. These rats received vehicle administration after the stressors in their test sessions.

### 3.3. Apparatus

The experiments were conducted using standard operant conditioning chambers (Coulbourn Instruments, Allentown, PA) housed in ventilated sound-attenuating cubicles (Coulbourn Instruments) and equipped with fans to mask outside noise. Two response levers were located on one wall of the chamber with a food trough located between the levers. Each lever was located 1 inch from the side wall with three “cue” lights positioned directly above it. Downward pressure (about 25 g) on a lever could result in a programmed consequence and was tabulated. Experimental chambers interfaced to a PC computer and a software program (Graphic State Notation; Coulbourn Instruments) was used to control the session parameters and collect data automatically.

### 3.4. Discrimination Training

Rats were trained to discriminate cocaine (10 mg/kg IP) from saline in a two-lever, food-reinforced operant task as described previously [42]. Rats were food-restricted to 85% of their free-feeding body weight and shaped to lever press for food pellets (45 mg; BioServ, Inc.) under a fixed ratio 1 (FR1) schedule of reinforcement. The availability to lever-press for food was signaled by the onset of the house lights in the experimental chamber and illumination of cue lights above both levers. For the training phase, which continued until 10 responses (FR10), completed on either lever, the rats were required to obtain a food pellet. When responding was stable, cocaine discrimination training began. On cocaine training days, cocaine (10 mg/kg, IP) was given, the rat was placed in the experimental chamber, and one lever was designated active. On vehicle training days, saline was administered (1 mL/kg, IP), the rat was placed in the experimental chamber, and the other lever was designated active. The training sessions began 15-min after injections were given at which time the house and cue lights were illuminated. Sessions terminated after 50 reinforcers were obtained or 15-min had elapsed, whichever occurred first. The designation of cocaine- and vehicle-appropriate levers was nonsystematic across rats. A double alternation sequence of training (Cocaine, Cocaine, Saline, Saline, Cocaine, *etc*.) was used 5 days/week until 90% or greater of the responses were on the injection-appropriate lever for four consecutive (2 Cocaine; 2 Saline) sessions. A nonsystematic order of training rats in experimental chambers was used to eliminate the possible effects of odor cues from previously trained rats [43].

### 3.5. Discrimination Testing

Once cocaine demonstrated stimulus control over behavior as defined, discrimination testing began. Exposure to each stress was limited to one time because multiple exposures to the same stress does not alter some behavioral effects of cocaine whereas multiple exposures to different stressors does alter these effects [44]. In order to limit the exposure to each stress to one time and obtain cocaine dose-response data in the present study, we utilized a cumulative dosing procedure [45]. Rats were given IP cocaine (1 mg/kg) injections and 15-min later placed in the experimental chamber for a 10-min test in which both levers were active (e.g., a food pellet was given after 10 presses on either lever) and a maximum of 10 reinforcers were made available. Like training sessions, these sessions ended after all available reinforcers were earned or after 10-min, whichever occurred first. At the end of this time, a second cocaine injection (2 mg/kg) was given and 5-min later, a second 10-min test performed. At the end of this time, a third injection (7 mg/kg) was given and 5-min later, a final 10-min test was performed. Thus, rats received cumulative doses of 1, 3, and 10 mg/kg cocaine.

To familiarize the animals with this 3-part testing procedure, we ran the cumulative session in which vehicle injections preceded each of the three segments of the session. Then, a baseline cumulative dosing session was run with cocaine injections. Finally, three test sessions with cocaine were run and these commenced immediately following the termination of one exposure to each of the three stressors described below.

### 3.6. Stressors

Three stressors were used in this study. For restraint stress, each animal was placed in a restraint cone (Harvard Apparatus, South Natick, Mass., USA) for 15-min. For the second stress, animals were given random foot-shock for 15-min (5 shocks of 5 mA; 100-msec duration) in an experimental chamber that was modified visually by placing a black and white striped panel in front of the wall containing the levers and food trough and utilizing flashing lights. The third stress was being placed in this context with no shock present on the day following four days of pairings of shock plus context. Stressors were presented in a nonsystematic order with the exception that the shock context test always followed the shock test. There was an interval of at least 7-days between test sessions with the exception of the shock and shock context test which had a 5-day interval. Discrimination training continued during these interim periods.

### 3.7. Corticosterone Assay

To determine whether the stressors employed in the present behavioral study activated HPA axis function, corticosterone levels were assessed in the cocaine test rats one or more weeks after the conclusion of the behavioral study. Blood samples were obtained by pricking the end of the tail and “milking” blood from it. Samples (about 0.5–1.0 mL) were collected on five separate occasions, 15-min after each of the three stressors (see above), 15-min post cocaine (10 mg/kg IP) injection, and 15-min post saline injection. This post-injection time was chosen because it corresponds to the initiation of the behavioral test sessions. Blood samples were taken at the same time of day as the behavioral sessions were conducted and this was performed in a nonsystematic order with at least a 3-day interval separating these sessions. Plasma was separated and corticosterone levels were assayed using RIA (ICN Biochemicals; Costa Mesa, CA). Blood samples were obtained from six of the eight F344 rats and from all eight of the Lewis rats.

### 3.8. Drugs

Cocaine hydrochloride was provided by the National Institute on Drug Abuse (NIDA, Research Triangle Institute, Research Triangle Park, NC). Cocaine was dissolved in sterile isotonic saline; cocaine solution and vehicle were administered IP in a volume of 1 mL/kg.

### 3.9. Data Analysis

Data on percent of cocaine-appropriate responses (cocaine-appropriate lever presses/total lever presses × 100) and rate of responding (total number of lever presses/session duration in sec) were analyzed using a 2 × 4 × 3 analysis of variance (ANOVA) with Strain (Lewis, F344) as a between-groups factor and repeated measures on Condition (Baseline, shock, restraint, shock-context) and Dose (1, 3, 10 mg/kg cocaine) or, in the case of the vehicle test groups, Segment (1–3). These analyses were followed-up with separate ANOVAs by strain. At least one response requirement (10 presses on one lever) must have been completed in order to be included in the analyses. Corticosterone levels were analyzed using a 2 × 4 analysis of covariance (ANCOVA) with Strain as a between-groups factor and repeated measure on Condition (cocaine, shock, restraint, shock-context) using the baseline condition (vehicle injection) as the co-variate. Significant effects were then followed up with separate 2 × 2 ANOVAs using the factors of Strain and Condition (Baseline *vs.* stress).

## 4. Conclusions

Results of the present study show that acute stress differentially alters the behavioral effects of cocaine in a strain- and stress-selective manner. Exposure to one type of stressor (restraint) enhances the discriminative stimulus effects of cocaine in F344 rats whereas exposure to the other stressors (shock and shock-paired context) enhances response rates to cocaine in Lewis rats. The enhancement in cocaine discrimination may reflect, in part, a restraint stress-induced generalization to cocaine as reported previously [38]. Indeed, in the present study, both F344 and Lewis rats in the vehicle test groups show increased cocaine-appropriate responding after restraint stress (Table 3) suggesting some substitution effects of restraint stress. However, the increase in such responding, although significantly greater than that seen under baseline (non-stress) conditions, was not at a level typically considered generalization or even partial generalization. Further, while vehicle test rats of both strains show increased cocaine-appropriate responding after restraint stress, only F344 rats show this effect among the cocaine test groups. It is also difficult to determine whether even a partial generalization to the cocaine discriminative stimulus after stress contributed to the enhanced effects seen in cocaine test rats because the vehicle test rats showed compromised responding after all stress exposures. Nonetheless, the data from the present study add to the literature that shows that stress can modulate the behavioral effects of psychoactive drugs. Like results from the present study, these effects appear to show specificity to the stressor, the test drug, and the strain and species tested [46].

Strain differences in baseline cocaine discrimination behavior may have worked against finding a stressor effect on discrimination behavior in Lewis rats. In this strain, the level of cocaine-appropriate responding at the lowest cocaine dose was somewhat higher than that seen in F344 rats that showed predominately vehicle-appropriate responding at this dose. Yet, the level of responding at the highest cocaine dose was nearly double that seen at the lowest dose under baseline conditions in this strain. On the other hand, the discriminative stimulus effect of cocaine may have been attenuated by stressor exposure in Lewis rats. Previous studies report that stressor exposures in the absence of cocaine resulted in full generalization to the cocaine- or amphetamine cue [38,39]. These results suggest stress exposure should have engendered greater degrees of cocaine-appropriate responding at the lower cocaine doses. While Mantsch & Goeders [38] found that restraint stress coupled with vehicle injections generalized fully to cocaine in the group of Wistar rats trained with the same cocaine dose as used in the present study, this effect was not significant in the group trained with 20 mg/kg cocaine. Although we did see evidence that restraint stress increased cocaine-appropriate responding in vehicle test rats, these results are limited due to the suppression of responding induced by the stress exposures. Nonetheless, restraint stress may affect the discriminative stimulus effects of cocaine in a non-linear dose-related manner, an effect that may differ by background strain.

There was also a strain-selective effect of stress on response rate after cocaine administration. Both footshock and the shock-paired context increased the rate of responding to cocaine in Lewis rats, while none of the stressors altered response rates in F344 rats. Other investigators observed large decreases in response rate following restraint stress in Wistar rats [38]. Yet, the stress of social defeat did not affect response rate to amphetamine or cocaine in Long Evans rats although discrimination behavior was affected [39]. Response rates decreased with increasing cocaine doses under fixed ratio schedules of responding, whereas low doses of cocaine were associated with increased response rates [47,48,49,50]. Thus, the stress-induced increases in response rates to cocaine seen in Lewis rats may reflect a decrease in cocaine sensitivity. Although this runs counter to the usual pattern of stress-induced increases in behavioral responses to psychoactive drugs (see Introduction), there may be a non-monotonic relationship, rather than a linear one, between stress or HPA activity and the behavioral effects of drugs [17].

Baseline corticosterone levels were somewhat higher in F344 compared to Lewis rats, a finding seen in previous studies [27,28,29], but this effect was not significant in the present study. Corticosterone levels increased above baseline for both strains after all stressors and after cocaine. In contrast to a prior study using immature, female rats [31], corticosterone levels reported in the present study that utilized adult, male rats did not show greater increases in F344 compared to Lewis rats after most stressors. In fact, cortiosterone levels increased to a greater extent in Lewis rats after shock compared to F344 rats. No strain differences were observed in cocaine-induced corticosterone levels, in contrast to previous work examining effects of cocaine [50] or morphine [51] administration on circulating corticosterone levels. These discrepancies may be due to methodological differences, for example, in time of day or route of drug administration.

Comparing corticosterone levels across stressor conditions and strains in conjunction with the behavioral results suggests that stress is not a unitary phenomenon. Within each strain, the stressor that altered behavior (discrimination or response rate) did not necessarily correspond to its effect on corticosterone levels. For example, the two stressors that increased corticosterone levels the most and within the same range as cocaine in F344 rats did not alter their behavior. Although shock exposure both increased corticosterone levels and response rates more in Lewis *vs.* F344 rats, exposure to the shock context increased corticosterone levels more in F344 than Lewis rats. Yet, this stressor affected behavior in Lewis rats but not in F344 rats. It is possible that behavioral or hormonal outcomes would differ if the levels of each stress were varied (e.g., longer restraint stress time or greater number of foot shocks delivered). That each stressor type was only tested under one set of parameters is a limitation of the present study.

In the present study, exposure to the shock-paired context increased the rate of responding to cocaine in Lewis rats, but did not alter either response rate or discrimination behavior in F344 rats. Yet, F344 rats showed a greater increase in corticosterone secretion in response to the shock–paired context over baseline compared to Lewis rats. Similarly, Glowa *et al.* [30] showed that Lewis rats exhibit a greater auditory startle response (e.g., unconditioned response) than F344 rats but F344 rats showed a greater corticosterone response to the startle context (e.g., conditioned response). Strain differences in conditioned responses to aversive stimuli have been shown. F344 rats showed greater anticipatory (conditioned) responding to escapable and inescapable shock [53], greater conditioned fear (freezing) in response to both the shock-paired context and to the discrete auditory CS paired with footshock [54], and greater fear-conditioned suppression of drinking [32] compared to Lewis rats. Further, although Lewis rats exhibited greater conditioned place preference to cocaine, F344 rats showed conditioned place aversion to similar cocaine doses [36]. F344 rats also emitted greater conditioned locomotor activity in a cocaine-paired context compared to Lewis rats [34]. DeVries *et al.* [55], who observed conditioned increases in corticosterone levels in response to exposure to the cocaine-paired locomotor context, suggest that conditioned responses in this paradigm reflect aversive conditioning. These data are therefore consistent with the notion that F344 rats show greater behavioral and neurohormonal reactivity to aversive conditioned stimuli than Lewis rats and that the range of stimuli perceived as aversive is greater in this strain, a hypothesis we are currently pursuing.
